# Study protocol: the relation of birth weight and infant growth trajectories with physical fitness, physical activity and sedentary behavior at 8-9 years of age - the ABCD study

**DOI:** 10.1186/1471-2431-13-102

**Published:** 2013-07-09

**Authors:** Arend W van Deutekom, Mai JM Chinapaw, Tanja GM Vrijkotte, Reinoud JBJ Gemke

**Affiliations:** 1 Department of Pediatrics, EMGO Institute for Health & Care Research, VU University Medical Center, Amsterdam, the Netherlands; 2 Institute for Cardiovascular Research VU, VU University Medical Center, Amsterdam, the Netherlands; 3 Department of Public and Occupational Health, EMGO institute for Health & Care Research, VU University Medical Center, Amsterdam, the Netherlands; 4 Department of Public Health, Academic Medical Centre, University of Amsterdam, Amsterdam, the Netherlands

**Keywords:** Developmental origins of health and disease, Birth weight, Child growth, Physical fitness, Muscle strength, Aerobic fitness, Physical activity, Sedentary behavior, Physical activity questionnaire

## Abstract

**Background:**

Low birth weight and accelerated infant growth have been identified as independent risk factors for childhood and adult obesity and cardiovascular disease. This led to the ‘Developmental Origins of Health and Disease’ (DOHaD) hypothesis, stating that environmental factors during pregnancy and early postnatal life affect disease risk in later life. There is growing evidence that perinatal factors may influence adult health through the programming of energy balance regulation, including sedentary behavior and physical activity. The present study focuses on the influence of birth weight and infant growth on physical fitness, physical activity and sedentary behavior in 8-9 year old children, as this might partly explain the higher obesity and cardiovascular risk associated with low birth weight and accelerated infant growth. In addition, this study provides the opportunity for a validation study of a linguistic and cross-cultural translated physical activity questionnaire compared to accelerometer data. This article describes the study protocol for this study.

**Methods/Design:**

This is a study embedded in the Amsterdam Born Children and their Development (ABCD) birth cohort. In 200 children of Dutch ethnicity, physical fitness, physical activity and sedentary behavior were assessed at age 8-9. We measured aerobic fitness using the 20 meter multistage shuttle run test, and neuromuscular fitness using the standing broad jump and handgrip strength test. Sedentary behavior and physical activity levels were measured using accelerometry. All children also completed a translated physical activity questionnaire, the scores of which will be compared to accelerometry data to assess the construct validity of the questionnaire in Dutch school-aged children.

**Discussion:**

This study will be the first population-based prospective cohort study to address the association of both prenatal and postnatal growth with physical fitness and objectively-assessed physical activity and sedentary behavior. This will contribute to a better understanding of the way perinatal growth relate to lifestyle and obesity in later life. The results may guide both future studies in the field of DOHaD, and public health strategies in the prevention of childhood obesity.

## Background

In the 1980s Barker and Hall reported a series of studies regarding the association between low birth weight and death from coronary heart disease, suggesting that prenatal environmental factors are involved in the aetiology of adult-onset diseases [1,2]. In the following decades numerous epidemiological studies substantiated a close association between low birth weight and an increased risk of developing adult diseases, including cardiovascular disease [3,4], stroke [5], type 2 diabetes mellitus [6], hypertension [7], adiposity [8], osteoporosis [9], polycystic ovarian syndrome [10], depressive disorders [11], and psychoses [12]. These observations led to the ‘Developmental Origins of Health and Disease’ (DOHaD) hypothesis [13]. This hypothesis proposes that the fetus adapts to a substrate-limited intrauterine environment with so called predictive adaptive responses, resulting in permanent changes in tissue differentiation and hormonal and metabolic set points. In this way programming results in irreversible adaptations which enhance survival during postnatal nutritional constraint, but may increase susceptibility to adult-onset diseases in a substrate-rich environment after birth.

As the number of DOHaD related studies grew, several important additions and refinements to the hypothesis were made. First, an important feature of the epidemiological observations is that adult disease risk is continuous within the normal range of birth weight, rather than being a pathological feature which is expressed below a critical cut-off point [14]. Second, in addition to the role of the intrauterine environment, more recent observations have also drawn attention to the significance of infant growth in the predisposition to adult disease. Accelerated growth in infancy increases risk of obesity [15], type 2 diabetes [16], hypertension [17] and cardiovascular disease [18]. Thus, the critical window for developmental responses may extend into early postnatal life. Last, subtle sex differences in the progression and development of adult diseases are observed, with female offspring being relatively protected from the adverse effects of perinatal malnutrition [19,20]. The cause of this sex difference remains unclear, but a protective effect of female sex hormones is proposed as an intriguing mechanistic explanation.

Obesity plays a critical role in the association between perinatal growth and adult disease, often preceding insulin resistance, dyslipidemia, and hypertension in children and young adults [21]. Given that obesity is fundamentally the result of an inadequate energy balance regulation, in which energy intake exceeds expenditure, its developmental origins cannot be explained by structural changes or neuroendocrine dysregulation alone [22]. Therefore, research on the pathogenesis of obesity has recently focused on the developmental origins of behavior that is closely related to energy expenditure and energy intake, i.e., feeding behavior, physical activity and sedentary behavior.

Physical activity (PA) and sedentary behavior are components of energy expenditure that vary considerably between persons as well as for a given person over time [23,24]. Sedentary behavior refers to activities that involve little energy expenditure, such as lying down, sitting, watching television, using the computer and other forms of screen based entertainment [25]. Sedentary behavior is thereby a distinct class of behaviors rather than being the absence of PA.

Both PA and sedentary behavior are of particular interest in the study of the developmental origins of energy balance regulation. Animal studies have shown that offspring of nutritionally impaired pregnancies are significantly less physically active than controls, and show increased sedentary behavior [26-28]. These effects on PA and sedentary behavior seem to originate from programming of the hypothalamic pathways which regulate energy homeostasis [29]. In humans, several clinical studies also identified low birth weight as a predictor for lower PA levels and more sedentary behavior in children and adults [30-34], although others failed to confirm this relationship [35-41], and there are suggestions that the association may be limited to very low birth weight individuals [32].

Physical fitness is generally defined as the ability to perform sports or occupations, and is bidirectionally related to PA [42]. As recent data indicates, regular PA improves physical fitness, and conversely, a high level of physical fitness promotes higher PA levels [43]. It is therefore apparent that unfit children are less inclined to be physically active, which has considerable potential to increase later disease risk. There are many dimensions to fitness, of which aerobic fitness (also known as cardiorespiratory endurance) and muscular strength are most consistently associated with physical performance and cardiovascular health [44-46]. Although fitness can be improved by regular PA, it is partly genetically determined, and perinatal influences may attenuate or strengthen this predetermined fitness level [47]. Indeed, several studies consistently found lower muscle mass and strength in lower birth weight adults and children [48]. For aerobic fitness, the findings are less robust. A number of studies found a significant positive association between birth weight and aerobic fitness in childhood and adulthood [34,49,50], but others did not [51,52]. Differences in subject age, sample size, type of fitness test used and severity of growth retardation in the cohort may explain these contradictory reports.

The current evidence suggests that physical fitness and PA may have a combined and accumulative effect on cardiometabolic health from an early age. Especially body fatness is inversely associated with fitness and PA levels [42]. But improved physical fitness and PA also promote a healthier cardiovascular risk profile in children independently from body fatness, including lower blood pressure, higher HDL cholesterol, lower triglycerides, improved glucose tolerance and post-prandial lipidemia, and modified clotting factors [42,53]. Therefore, variations in physical fitness and PA might impact future health of children irrespective of obesity risk.

In recent years, sedentary behavior has been identified as a distinct health risk factor. For example, sedentary behavior has been shown to be positively associated with an increased risk of type 2 diabetes, cancer, and all-cause and CVD mortality in adults [54-56]. These associations are shown to be at least partially independent of levels of PA. Reviews of the relationship between sedentary behaviors and obesity in children and adolescents found a positive association, suggesting that sedentary behavior is a risk factor for the development of obesity in children [57,58]. Another review, however, concluded there was insufficient evidence for a longitudinal relationship between childhood sedentary time and cardiovascular health indicators, because of a lack of high quality studies addressing this topic [59].

**Figure 1:**
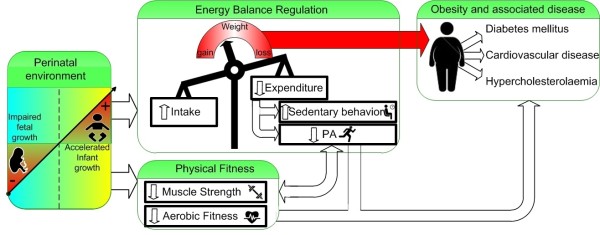
**Hypothesized model of the perinatal influences on later disease through energy balance and fitness.** Graphical representation of the hypothesized associations of the perinatal environment with obesity and associated diseases through physical fitness, physical activity and sedentary behavior Abbreviations: *PA*, Physical activity.

Based on the aforementioned data, we hypothesize that low birth weight and accelerated infant growth is associated with lower levels of aerobic fitness, muscular strength and PA and more time spent on sedentary behavior in school aged children, contributing to the increased propensity to obesity and related diseases in later life. The hypothesized model is shown graphically in Figure 1.

This paper describes the rationale and design of a study aiming to examine the association of birth weight and infant growth trajectories with aerobic fitness, muscular strength and objectively assessed sedentary behavior and PA levels in 8-9 year old children. In order to assess whether an accurate assessment of regular PA can be obtained by self-report questionnaires in Dutch children, we will additionally validate a linguistic and cross-cultural translation of a commonly used PA questionnaire in 8-9 year old children. If validity of this questionnaire has been confirmed in our subgroup, we may use it to assess PA and sedentary behavior of our entire cohort in a future phase of the ABCD study. This article outlines the development of this PA questionnaire.

### Study objectives

The primary study objectives are to examine the association of birth weight and infant growth trajectories with (i) aerobic fitness assessed by the 20 meter multistage shuttle run test (20-m MSRT), (ii) muscular strength assessed by the standing broad jump (SBJ) and hand grip strength test, and (iii) physical activity levels and sedentary time assessed by accelerometry, at 8 to 9 years of age. We will control for potential confounders, such as maternal factors (e.g., maternal age, pre-pregnancy body-mass index (BMI), maternal height, socio-economic status) and child factors (e.g., gender, age, height, BMI, gestational age, Apgar score), and test for possible effect modification by gender. (see Table 1) A secondary objective is to examine the construct validity of a self-report extensive PA questionnaire, based on the internationally employed Physical Activity Questionnaire for Older Children (PAQ-C) and Children’s Leisure Activities Study Survey (CLASS). This PA questionnaire is provided in the appendix (see Additional file 1).

**Table 1:** Overview of study variables

***Study outcome *construct**	**Component**	**Test(s) (unit)**	***Determinants *construct**	**Variable(s) (unit)**	***Potential confounders *Variable(s) (unit)**
**Physical fitness**	Aerobic fitness	20 meter multistage shuttle run test (stage)	**Intra-uterine growth**	Birth weight (SD)	**Subject-specific:**
					• Gender (male/female)
					• Age (years)
					• Height (cm)
					• BMI (kg/m^2^)
					• Gestational age (days)
					• Apgar score (score at 5 min)
	Muscular strength	Hand grip strength test (kg) Standing Broad Jump test (cm)	**Infant growth**	Average standardized growth velocity 0-12 months (ΔSDS)	**Family-specific:**
					• Maternal age (years),
					• Maternal height (cm)
					• Pre-pregnancy BMI (kg/m^2^),
					• Socio-economic status (class)
**Physical activity**	Moderate to vigorous physical activity	Accelerometry (minutes/day)			
		PA questionnaire (score)			
**Sedentary behavior**	Sedentary time	Accelerometry (minutes/day)			
		PA questionnaire (score)			

Overview of the primary study outcomes (left column) and determinants (middle column). Described are the studied constructs with the corresponding tests and units of measurement. The right column displays the potential confounders we will control for. Abbreviations: *kg*, kilograms; *cm*, centimeters; *SD*, Standard Deviations; *ΔSDS*, Difference in Standard Deviation Scores.

## Methods/Design

### Setting

This study is embedded in the Amsterdam Born Children and their Development (ABCD) study, a prospective birth cohort study aimed at the identification of prenatal and early life influences on health at birth and in later life. The project is carried out by a multidisciplinary team of researchers from the Public Health Service Amsterdam, the Academic Medical Center and VU University Medical Center.

The design and conceptual framework of the overall ABCD-study has been previously described in detail [60]. In summary and relevant to the current study, the ABCD-study included 8266 women pregnant between January 2003 and March 2004. These woman completed questionnaires during and after their pregnancies, covering sociodemographic data, lifestyle, dietary habits and psychosocial factors, conventional medical and obstetric history, and perinatal details. Birth weight of the children was obtained from the Child Health Care Registration, the office that also performs neonatal blood screening for inborn errors of metabolism, as is routinely performed in the Netherlands in the 1^st^ week of life. Until the age of 4 years, height and weight measures of these children were routinely collected as part of regular preventive Child Health Care. These measurements took place during on average 14 regular follow-up moments and were performed by qualified nurses and physicians. Of the 6735 women who gave permission for follow-up of their child, 6575 mothers also consented to follow up of their child’s growth data.

In 2008, around their 5^th^ birthday, 3321 children completed a ‘health check’. At the appointment for this health check we collected data on anthropometrics, blood pressure, heart rate variability, body composition and a capillary blood sample to determine lipid profile, glucose, insulin, C-peptide, HbA1c and albumin. In addition, parents completed questionnaires about the child’s health, development and behavior, family socio-demographics, maternal lifestyle and psychosocial conditions and family history of medical conditions.

### Birth weight and infant growth

The primary determinants in our study are birth weight and infant growth trajectories. Birth weight is hereby used as a surrogate marker for intrauterine growth, and an established index for the intrauterine environment.

Birth weight is expressed as standard deviation (SD) score, adjusted for maternal height, weight, parity, ethnicity and fetal sex, in accordance with the recommendations of Gardosi [61]. Low birth weight is defined as a birth weight below the 10^th^ percentile (-1.28 SD). Infant growth is expressed as the average standardized growth velocity, defined as the change in SD score between birth and 12 months. We will examine growth in weight-for-age, height-for-age and weight-for-height. We expect the association with the outcome measures to differ for each expression of growth, with the strongest association for weight-for-age. Accelerated growth is defined as >0.67 change in SD score. This 0.67 SD represents the distance between two adjacent growth lines on standard infant and childhood growth charts (i.e., the 2^nd^, 10^th^, 25^th^, 50^th^, 75^th^, 90^th^ and 98^th^ centile lines). The adopted definitions of low birth weight and accelerated infant growth are based on recommendations of Monteiro et al. [15], and in line with recent other studies addressing the DOHaD hypothesis [62-65].

### Current study population

The current study is conducted in a subgroup of the ABCD-birth cohort. At the time of the data collection, the children were 8 or 9 years of age. Children were eligible for this study if (i) they fully completed the ABCD age 5 health check and (ii) were of Dutch ethnicity (i.e. with both parents born in the Netherlands). This is to exclude confounding by ethnicity. Exclusion criteria were (i) a personal history of neurological, cardiovascular or metabolic disease, (ii) any musculoskeletal injury or disability or (iii) the use of any medication at the time of data collection.

### Sample size calculation

Based on the literature, we expected to find a difference between low birth weight and normal birth weight children of 1 stage on the 20-m MSRT, with a SD of 2 stages [49,66], and a difference of 10 cm on the SBJ test, with a SD of 25 cm [67]. A similar difference in 20-m MSRT and SBJ test scores between children with normal and accelerated infant growth was anticipated, but as there is no literature on this topic available, a scientific basis for this assumption is lacking. With a significance level of 5% and a power of 80% in total 100 children per group were required.

### Recruitment

To ensure an adequate number of children in each subgroup, we selected more children with low birth weight and/or accelerated growth than that would be selected by random sampling. This to ensure power of the study that may be lost in a more generalized sampling technique.

A recruitment letter, explaining study objective and design in detail, was sent to all children´s parents. For the children, simplified written information was provided. Informed and written consent were obtained from parents before participation. The Amsterdam Medical Center’s Ethics Committee approved the study protocol, and all procedures were performed in accordance with the ethical standards of the Helsinki Declaration of 1975 as revised in 2008 [68].

### Study design

Three identical measurement periods were arranged two or three weeks apart. Each measurement period consisted of (A) the self-completion of a PA questionnaire, (B) a physical fitness test battery, and (C) wearing an accelerometer for 7 consecutive days.

### A. Modified physical activity questionnaire

After written consent was obtained, a PA questionnaire was sent to the children by surface mail. The questionnaire was completed at home and handed back at the day of the physical fitness test battery. Responses were checked for completeness by staff.

The PA questionnaire is a cross-cultural adaptation of the Physical Activity Questionnaire for Older Children (PAQ-C) [69] and the Children’s Leisure Activities Study Survey (CLASS) [70]: two standardized activity questionnaires for children, both of which recall activities over the previous 7 days.

The PAQ-C is a 7-day self-reported recall instrument, developed to assess general levels of PA throughout the elementary school year for children approximately 8 to 14 years of age [71]. It provides a summary PA score derived from nine items. Each question is scored on a 5-point scale, with higher scores indicating higher levels of activity. The first question is a checklist of 22 common leisure and sports activities. Because the PAQ-C only assesses the frequency of activities, we added elements of the CLASS addressing duration of the activities. For each PA in the checklist, children were asked to report the frequency and duration spent in that activity. The next six items addresses PA during physical education classes, recess, lunch break, right after school, in the evenings, and on the weekend. Item eight asks which statement “describes you best for the last 7 days” with five statements describing low to very high activity levels. The ninth item asks the child how often he/she participated in PA on each day of the week [69].

The PAQ-C is widely accepted [72-74] and recommended [75] for international and national studies. In addition, a systematic review of measurement properties of self-report PA questionnaires for children concluded good to moderate validity and reliability of the PAQ-C [76]. The CLASS questionnaire was found to have weak to moderate validity and reliability. Nonetheless, both the PAQ-C and CLASS questionnaire were considered amongst the most promising PA questionnaire for children [76].

#### Translation of the physical activity questionnaires

The aim of the translation process was to develop a Dutch version of the PAQ-C that is conceptually equivalent but adapted to represent common Dutch physical activities. The translation process was carried out using a forward-backward translation technique [77-79]. First, three researchers, native Dutch speakers and fluent in English, produced independent forward translations of the original PAQ-C and CLASS questionnaire. Then, the researchers and the project manager produced a reconciled Dutch version based on the forward translations and the original questionnaires, and an English report documenting the synthesis process, the issues addressed, and how they were resolved. Then, a native English and fluently Dutch speaking translator, who was blind to the original version, translated this reconciled Dutch version back into English. One of the researchers and the project manager compared the back-translated version with the original version, assessing inaccuracies, misunderstandings or mistranslations, and produced a prefinal version. The project manager and all involved researchers replaced PA items that are rarely conducted by Dutch children (e.g., Australian football, cross-country skiing) by items that are more commonly practiced (e.g., playing tennis, badminton, practicing judo). This version was administered to 10 Dutch children of primary school age (i.e., the target population) checking the comprehensibility of each item. As there were no ambiguities reported, we concluded that the adapted version retained its equivalence in an applied situation. The final PA questionnaire used in this study is provided in the Appendix (see additional file 1).

### B. Physical fitness test battery

A physical fitness test battery was developed using the evidence-based recommendations of the ALPHA health-related fitness test battery for children and adolescents [80] and the Eurofit Fitness test Battery [81]. We successively assessed anthropometry, blood pressure, maximum muscle strength, explosive muscle strength and aerobic fitness.

#### Anthropometry

Height and weight were measured with the children dressed in light sportswear. Height was measured to the nearest millimetre using a Leicester portable height measure (Seca), and weight to the nearest 100 gram using a Marsden weighing scale, model MS-4102.

#### Blood pressure

Blood pressure was measured two times on the right arm in a sitting position after a few minutes rest. If the difference between the measurements was more than 10 mmHg for either systolic or diastolic pressure, a third measurement was obtained. The device used was the Omron 705 IT (Omron Healthcare Inc, Bannockburn, IL, USA) with an appropriate sized cuff. Outcome measures were systolic pressure (SP), diastolic pressure (DP), and mean arterial pressure (MAP), all expressed in mmHg. MAP was calculated using the following equation: MAP = DP + 1/3 (SP-DP) [82].

#### Muscular strength

With the standing broad jump (SBJ) and hand grip strength test we assessed explosive muscle strength and maximum muscle strength, respectively.

In the SBJ test, the child attempts to jump as far as possible with feet together. From a starting position immediately behind a line with feet slightly apart, the child jumps using both feet with take-off and landing, swinging the arms and bending the knees to provide forward drive. The distance was measured from the take-off line to the point where the back of the heel nearest to the take-off line lands, and reported in centimetres. The test was repeated twice, and the best score retained.

Hand grip strength was measured using a hand dynamometer, with the scores recorded in kilograms (kg). The reported precision is 0.5 kg. The test was performed twice (alternately with both hands), with the dynamometer adjusted to the age- and gender-specific optimal grip span, as this seems the most appropriate protocol to evaluate maximal hand grip strength in children [83].

The SBJ test seems to be the most valid test assessing lower body muscular strength compared to other muscular strength tests (i.e., bent and extended arm hang, squat jump, countermovement jump, Abalakov jump), showing the highest associations with isokinetic parameters [80]. In addition, Castro-Piñero et al. concluded that the SBJ test can be considered a general index of lower body muscular fitness in children, based on a strong association with other lower body muscular strength tests (R^2^ = 0.83–0.86), as well as with upper body muscular strength tests (R^2^ = 0.7-0.9) [67]. Milliken et al. analysed the association between hand grip strength and chest press in children aged 7-12 years and found that the hand grip strength test is valid to assess upper body maximal strength (R^2^ = 0.70) [84].

#### Aerobic fitness

We measured aerobic fitness using the 20 meter multistage shuttle run test (20-m MSRT); a simple non-invasive, valid and reliable field test providing an estimate of maximal cardiorespiratory capacity [85]. The test was conducted as described by Léger et al. [86] On an outdoor field, children were required to run between two lines 20 m apart, while keeping pace with beeps emitted from a pre-recorded CD. The initial speed was set at 8.5 km • h^-1^ (2.4 m • s^-1^), increasing by 0.5 km • h^-1^ (0.1 m • s^-1^) with each stage thereafter (one stage takes one minute). Children were instructed to run in a straight line, to pivot and turn on completing a shuttle, and to pace themselves in accordance with the beeps. The test ended when the child failed to reach the end line concurrent with the beeps on two consecutive occasions. The children were verbally encouraged by the researchers to run for as long as possible throughout the course of the test. The last completed half-stage of the 20-m MSRT was recorded and used as a valid proxy for aerobic fitness [87,88].

The 20-m MSRT has several advantages over other field tests, such as tests that cover as much distance as possible in a set time [89], or tests that cover a set distance in the fastest time possible [90]. First, the 20-m MSRT has a graded physiological response and the absence of individual pace control [91,92]. Second, it can be administered in a relatively small space and is therefore easy to implement without extensive facilities. Third, children are known for their frequent stops, starts, and turns in their daily PA. Therefore, it may be a more relevant test than a continuous directional run.

### C. Accelerometry

Accelerometers have shown to provide reliable estimates of overall PA, sedentary behavior and PA-related energy expenditure among children [93,94]. In this study, PA and sedentary behavior were objectively assessed using two models of Actigraph (ActiGraph, LLC, Fort Walton Beach, FL) accelerometers, namely triaxial Actitrainers (dimensions: 8.6 cm × 3.3 cm × 1.5 cm, weight: 51 grams) and GT3Xs (dimensions: 3.8 cm × 3.7 cm × 1.8 cm, weight: 27 grams). These are omnidirectional accelerometers, sensitive to movement in all directions.

Researchers distributed the accelerometers face-to-face at the end of the physical fitness test battery. Information about accelerometer use was given to the children and parents orally. Researchers placed the accelerometer to the children’s waist using an elastic waistband, and they told the children not to remove the device for 7 days except during sleeping, swimming and bathing. Additionally, children and parents received a brochure with information about accelerometer use and a compliance log. Children had to self-complete this compliance log for the duration of the accelerometer data collection, as this is an effective, low cost method to increase compliance [95]. Each day, the children recorded the time they got up and went to bed, whether or not it was a school day, and whether the accelerometer was removed during the day, and if so, for what reason. After the proposed wearing period, the devices were collected at the child’s home by the researchers. Downloading the data from accelerometers was done as soon as possible on the same computer where it was initialized to prevent disturbances that can be caused by the time offset between computers [96].

The accelerometer signal is summarized over a user-defined period of time, called epoch, into what are called counts. The higher the number of counts, the higher the intensity of PA. As children tend to have short bursts of PA [97], we selected an epoch length of 15 seconds to accurately capture this spasmodic PA pattern [98]. Non-wearing time was defined as more than 20 minutes of consecutive zero counts. Wearing time was calculated by subtracting non-wearing time from 24 hours. To capture a representative portion of daily PA, only days with at least 10 hours of wearing time were considered valid [99]. Children who had at least three valid weekdays and one valid weekend day were included in further data analysis [100]. Based on the cut-off values of Treuth et al. we defined minutes spent in sedentary, light, moderate and vigorous activity, as <100, 100 – 2999, 3000 – 5199 and ≥5200 counts per minute (c.p.m.), respectively [101].

### Statistical analysis

Data will be presented as means ± SD for continuous variables, and as percentages for categorical variables.

For every primary outcome, its association with standardized birth weight and infant growth will be assessed using multivariable linear regression analysis. First, birth weights and infant growth trajectories will be dichotomized as normal vs. low birth weight and normal vs. accelerated infant growth, respectively, and analyzed together in a linear regression analysis to account for mutual interference. To assess whether the effect of low birth weight and accelerated infant growth exceeds the sum of each exposure separately, we will subsequently add an interaction term of birth weight with infant growth to the model. A p-value less than 0.10 for the interaction term is considered indicative of effect modification, after which a subgroup analysis will be performed with the children divided into four subgroups (normal birth weight and normal infant growth; low birth weight and normal infant growth; normal birth weight and accelerated infant growth; low birth weight and accelerated infant growth).

A scatterplot will be employed to identify potential linear associations of either birth weight or infant growth with the outcome variable. If the scatterplot suggests a linear association of either birth weight or infant growth with the outcome, we will additionally run the analysis with the respective dichotomized variable replaced by its continuous equivalent.

The model will be adjusted for potential confounding, by addition of the potential confounders group-wise in a multivariable linear regression analysis. The first group of potential confounders are subject-specific variables (gender, current age, height, BMI, gestational age, Apgar score), the second are family-specific variables (socio-economic status (defined as maternal level as education), maternal age, maternal height, maternal pre-pregnancy BMI). Effect modification by gender will be evaluated by addition of an interaction term of gender with either birth weight or infant growth.

### Validity assessment of the questionnaire

To assess the construct validity of the modified PA questionnaire, PAQ-C summary scores will be examined as originally constructed (the average of the nine questions) [69]. Pearson’s correlation coefficient between the PAQ-C summary score and accelerometer-assessed moderate to vigorous PA (MVPA) will be calculated, to assess the construct validity of the PA questionnaire compared to accelerometry. In addition, the common leisure and sports activities assessed in the first question are assigned metabolic equivalent units (METs) based on the MET values given in the recently updated 2011 Compendium of Physical Activities [102]. These activities are classified as sedentary (<2 MET), light (2-4.5 MET), moderate (4.6-6.5 MET) or vigorous (more than 6.5 MET) PA, and grouped together to assess the daily time spent in each PA level. Agreement between self-reported minutes in MVPA and minutes in MVPA according to accelerometry data will be examined using the Bland–Altman procedure [103]. A similar approach will be used to assess the agreement between self-reported and accelerometry assessed sedentary time. The Bland-Altman procedure plots the differences between the self-reported and accelerometry data against their mean, where ±1.96 SD of the differences provides an interval within which 95% of the differences between the two sets of measurements are predicted to fall. A higher level of agreement is illustrated by a mean difference closer to zero and a smaller range of the 95% confidence interval.

## Discussion

Although low birth weight and accelerated infant growth have been consistently linked to childhood and adult obesity [8,15], the underlying disturbances in energy balance regulation still remain poorly understood. The present study focuses on the developmental origins of physical fitness, physical activity and sedentary behavior in 8-9 year old children, as this might partly explain the higher obesity and cardiovascular risk associated with low birth weight and accelerated infant growth. In addition, this study provides the opportunity for a validation study of a linguistic and cross-cultural translated PAQ-C and CLASS questionnaire compared to accelerometer data. If the validation study suggests that the translated questionnaire is a valid tool to assess PA and sedentary behavior in this subgroup, it may be used in a later stage of the ABCD study, to assess PA and sedentary behavior in our entire birth cohort.

To our knowledge, this study is the first prospective cohort study addressing the association of both birth weight and infant growth with objectively assessed PA and sedentary behavior, aerobic fitness and muscular strength in later childhood. A major strength of this study is that it is conducted within the framework of the ABCD study, a prospective cohort study with extensive data on birth outcome, growth patterns, and lifestyle factors, amongst others. This provides the opportunity to control for a broad range of potential confounders, like BMI, gestational age, Apgar score, maternal age, maternal pre-pregnancy BMI, and socio-economic status. All these variables are prospectively collected and assessed during the course of the ABCD study [60].

A limitation of this study is that only children of Dutch ethnicity are included, to exclude confounding by ethnicity. As there are known ethnic differences in both obesity risk [104,105] and PA levels and sedentary behavior [106,107], the results might not be applicable to other ethnic groups. In addition, selective nonresponse in this study may lead to an oversampling of physically fit children, as these children might be more willing to participate in a study on PA and fitness. Both these aspects hamper the generalizability of the results.

In this study design, and in many others, birth weight below the 10^th^ percentile is used to identify intrauterine growth retardation [108]. However, this is only a very crude indicator [109], and there is increasing evidence that insults during fetal life can have long-term consequences independently of birth weight [110]. On the other hand, a low birth weight subject could be small at birth because of genetic reasons or maternal characteristics, and indeed have had a perfectly well-nourished fetal life. The use of customized growth standards to identify low birth weight subjects, in line with Gardosi’s recommendations [61], may partly avoid misclassification. Nonetheless, instead of a ‘present’ or ‘absent’ definition of low birth weight, it is considered more informative to assess whether there is a continuous association of outcome with birth weight [110]. In fact, substantial epidemiological data indicate there is a continuous inverse association of birth weight with certain chronic diseases [7,9,111]. In line with this, if we find a linear association of birth weight and/or infant growth with the outcome measure, we will report the results of linear regression analyses with birth weight both dichotomized as well as a continuous variable.

While the significance of infant growth in the predisposition to adult disease has extensive support from observational studies, it is not clear whether the detrimental adult outcomes (obesity and cardiovascular disease) are related to a specific time window. Different studies vary widely in definition and duration of accelerated growth, with the period of growth evaluated being as short as 1 week [112] up to the first 7 years of life [18]. A consistent age interval used in several studies was 0-1 year and 0-2 years, all of which also used the +0.67 weight-for-age SD score variation cut-off point as a definition for accelerated growth [15]. In line with the recommendations [15] and recent other studies [62-65], we will adopt the 0-1 year age interval and the 0.67 SD score variation for the period of infant growth and the definition of rapid growth, respectively.

There are consistent relations between PA and fitness status in adults and children [42]. Physical fitness is in part determined by PA patterns over recent weeks or months, although the improvement in fitness to a standard exercise dose varies widely and is considered predisposed [113]. It is stated that such individual predisposition reflects genetic traits [47], but the programming of PA patterns based on early life experiences offers an alternative explanation. Conversely, higher levels of physical fitness, especially aerobic fitness, may promote higher PA levels. As sports and active play may be more easy, successful and rewarding for the physically fit, these children may engage in PA more often. This poses individuals at the lower end of the activity and fitness distribution at considerable risk of a downward spiral of inactivity leading to reduced fitness and reduced fitness leading to less activity. Given the strong and consistent inverse relations of physical fitness and physical activity with obesity and cardiovascular risk profile, this will have detrimental effects on later health [42,53].

If the present study indeed shows that physical fitness, PA and sedentary behavior are significantly associated with birth weight and infant growth, further assessment of the potential mechanisms underlying these associations is required. For example, animal studies provide evidence that sympathetic nervous system (SNS) function is likely to be involved in the developmental origins of later obesity risk. Perinatal insults have been reported to alter SNS development, which may persist throughout life [114]. In human studies, an inverse association between birth weight and SNS activity in middle-aged adults [115], young adults [116] and adolescents [117] was found, although a study in neonates born small for gestational age found no such association [118]. SNS hyperactivity may contribute to obesity through increased food intake [119], altered glucose metabolism [120], and decreased energy expenditure [121]. Whether these developmentally-induced variations in SNS function influence PA and fitness remains to be investigated. In addition, alterations in body composition are widely believed to contribute to the pathogenesis of obesity and its complications. Low birth weight has been linked to lower adult and childhood muscle mass [48] and altered muscle metabolism [122]. To what extent these developmental influences on skeletal muscle mass and function explain variations in physical fitness and activity needs further investigation.

In conclusion, this study examines developmentally-induced variations in physical fitness, PA and sedentary behavior in children aged 8-9 years and contributes to an improved understanding of the perinatal influences on energy balance regulation. This knowledge may guide future studies in the field of developmental origins of obesity and related diseases. In addition, the results might initiate preventive public health strategies, as the early promotion of an active lifestyle may be an effective and efficient way to attenuate later disease risk in low birth weight and infant growth accelerated children.

## Abbreviations

ABCD: Amsterdam born children and their development; BMI: Body mass index; CLASS: Children’s leisure activities study survey; c.p.m.: Counts per minute; DOHaD: Developmental origins of health and disease; DP: Diastolic pressure; kg: Kilograms; MAP: Mean arterial pressure; MET: Metabolic equivalent units; MVPA: Moderate to vigorous physical activity; PA: Physical activity; PAQ-C: Physical activity questionnaire for older children; SBJ: Standing broad jump; SD: Standard deviation; SNS: Sympathetic nervous system; SP: Systolic pressure; 20-m MSRT: 20 meter multistage shuttle run test.

## Competing interests

The authors declare that they have no competing interests.

## Authors’ contributions

AD: Conception and design of study. Drafting the article and revision on critically important intellectual content. Final approval of the manuscript version to be published. MC: Conception and design of study. Drafting the article and revision on critically important intellectual content. Final approval of the manuscript version to be published. TV: Conception and design of study. Establishing and coordinating the ABCD-birth cohort. Drafting the article and revision on critically important intellectual content. Final approval of the manuscript version to be published. RG: Conception and design of study. Establishing the ABCD-birth cohort. Drafting the article and revision on critically important intellectual content. Final approval of the manuscript version to be published. All authors read and approved the final manuscript.

## Pre-publication history

The pre-publication history for this paper can be accessed here:

http://www.biomedcentral.com/1471-2431/13/102/prepub

## Supplementary Material

Additional file 1 **PA Questionnaire.** Physical Activity Questionnaire. Physical Activity Questionnaire as used in the study, based on the Physical Activity Questionnaire for Older Children (PAQ-C) and Children’s Leisure Activities Study Survey (CLASS), cross-culturally adapted to represent common Dutch physical activities. Click here for file
