# Supplementary material for: Study protocol: the relation of birth weight and infant growth trajectories with physical fitness, physical activity and sedentary behavior at 8-9 years of age - the ABCD study
Source: BMC Pediatr. 2013 Jul 9;13:102. doi: 10.1186/1471-2431-13-102 (PMC3710272; doi:10.1186/1471-2431-13-102)

Date of birth: …
Identification number: …

Physical Activity Questionnaire

We are trying to find out about your level of physical activity from ***the last 7 days*** (in the last week). This includes sports or dance that make you sweat or make your legs feel tired, or games that make you breathe hard, like tag, skipping, running, climbing, and others.

Remember: **1. There are no right and wrong answers — this is not a test.**

2. Please answer all the questions as honestly and accurately as you can — this is very important.

Do you make a mistake when filling out the questionnaire? Color the box all black and encircle it. Like this: ■. Then tick the appropriate box.

Good luck completing the questionnaire!

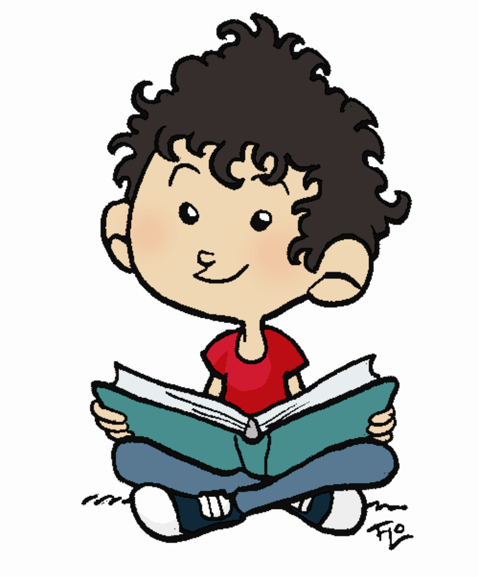

[Identification number.: #]

Physical activity in your spare time:

1. Have you done any of the following activities in the past 7 days (last week)? If yes, how many times and how long?

Have you done any of the following sports in the past 7 days (last week)? *Tick NO or YES*			If yes: How many times have you done the sport in the past 7 days (last week), and how long each time?	
			How many times?	How long each time?
Soccer on a sports club	NO ☐	YES ☐		
Soccer on the streets or in a playground	NO ☐	YES ☐		
Tennis on a sports club	NO ☐	YES ☐		
Tennis on the streets or in a playground	NO ☐	YES ☐		
Hockey on a sports club	NO ☐	YES ☐		
Hockey on the streets or in a playground	NO ☐	YES ☐		
Basketball on a sports club	NO ☐	YES ☐		
Basketball on the streets or in a playground	NO ☐	YES ☐		
Gymnastic sports	NO ☐	YES ☐		

Have you done any of the following sports <u>in the past 7 days (last week)</u>? *Tick NO or YES*			If yes: How many times have you done the sport <u>in the past 7 days (last week)</u>, and how long each time?	
			How many times?	How long <u>each time</u>?
Baseball or softball on a sports club	NO ☐	YES ☐		
Baseball or softball on the streets or in a playground	NO ☐	YES ☐		
Table tennis on a sports club	NO ☐	YES ☐		
Table tennis on the streets or in a playground	NO ☐	YES ☐		
Badminton on a sports club	NO ☐	YES ☐		
Badminton on the streets or in a playground	NO ☐	YES ☐		
Volleyball on a sports club	NO ☐	YES ☐		
Volleyball on the streets or in a playground	NO ☐	YES ☐		
Netball on a sports club	NO ☐	YES ☐		
Netball on the streets or in a playground	NO ☐	YES ☐		

Have you done any of the following sports <u>in the past 7 days (last week)</u>? *Tick NO or YES*			If yes: How many times have you done the sport <u>in the past 7 days (last week)</u>, and how long each time?	
			How many times?	How long <u>each time</u>?
Handball on a sports club	NO ☐	YES ☐		
Handball on the streets or in a playground	NO ☐	YES ☐		
Martial arts (such as judo, karate, kickboxing)	NO ☐	YES ☐		
Dance (such as ballet, street dance, jazz ballet)	NO ☐	YES ☐		
Ice skating	NO ☐	YES ☐		
Athletics	NO ☐	YES ☐		
Swimming laps	NO ☐	YES ☐		
Swimming for fun	NO ☐	YES ☐		
Cycling	NO ☐	YES ☐		
Other sports? Please state …………………………………………………………				

Have you done any of the following activities <u>in the past 7 days (last week)</u>? *Tick NO or YES*			If yes: How many times have you done the sport <u>in the past 7 days (last week)</u>, and how long each time?	
			How many times?	How long <u>each time</u>?
Playing tag	NO ☐	YES ☐		
Skipping rope	NO ☐	YES ☐		
Bounce on the trampoline	NO ☐	YES ☐		
Play on playground equipment	NO ☐	YES ☐		
Play in the cubby house	NO ☐	YES ☐		
Jogging or running	NO ☐	YES ☐		
Roller blading	NO ☐	YES ☐		
Skateboarding	NO ☐	YES ☐		
Scooter	NO ☐	YES ☐		

[Identification number.: #]

Have you done any of the following activities in the past 7 days (last week)? *Tick NO or YES*			If yes: How many times have you done the sport in the past 7 days (last week), and how long each time?	
			How many times?	How long each time?
Play with pets	NO ☐	YES ☐		
Walk the dog	NO ☐	YES ☐		
Bike riding in your free time	NO ☐	YES ☐		
Physical education class	NO ☐	YES ☐		
Sport class at school	NO ☐	YES ☐		
Travel by walking to school<br>(to and from school = 2 times)	NO ☐	YES ☐		
Travel by cycling to school<br>(to and from school = 2 times)	NO ☐	YES ☐		

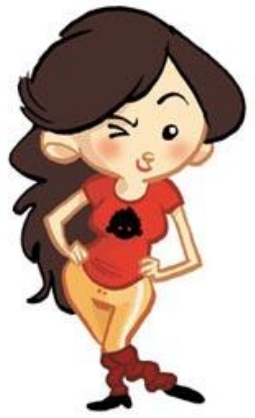

Have you done any of the following activities <u>in the past 7 days (last week)</u>? ***Tick NO or YES***			If yes: How many times have you done the sport <u>in the past 7 days (last week)</u>, and how long each time?	
			How many times?	How long <u>each time</u>?
Watching TV / videos / DVD	NO ☐	YES ☐		
Playing Playstation / Nintendo / Wii / DS / XBOX / computer games	NO ☐	YES ☐		
Other computer / Internet use	NO ☐	YES ☐		
Homework	NO ☐	YES ☐		
Playing indoors with toys	NO ☐	YES ☐		
Sitting talking	NO ☐	YES ☐		
Talk on the phone	NO ☐	YES ☐		
Listen to music	NO ☐	YES ☐		
Playing a musical instrument	NO ☐	YES ☐		
Playing board games / card games	NO ☐	YES ☐		

Have you done any of the following activities in the past 7 days (last week)? *Tick NO or YES*			If yes: How many times have you done the sport in the past 7 days (last week), and how long each time?	
			How many times?	How long each time?
Reading	NO ☐	YES ☐		
Art & craft (such as pottery, sewing, drawing)	NO ☐	YES ☐		
Household chores (such as dishwashing, vacuuming, washing the car)	NO ☐	YES ☐		
Imaginary play	NO ☐	YES ☐		
Travel by car/bus to school (to and from school = 2 times)	NO ☐	YES ☐		

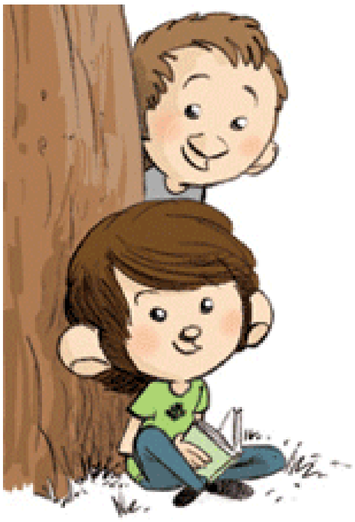

2. In the last 7 days, during your physical education (PE) classes, how often were you very active (playing hard, running, jumping, throwing)? (Check one only.)

☐ I don’t do PE

☐ Hardly ever

☐ Sometimes

☐ Quite often

☐ Always

3. In the last 7 days, what did you do most of the time at recess? (Check one only.)

☐ Sat down (talking, reading, doing schoolwork)

☐ Stood around or walked around

☐ Ran or played a little bit

☐ Ran around and played quite a bit

☐ Ran and played hard most of the time

4. In the last 7 days, what did you normally do at lunch (besides eating lunch)? (Check one only.)

☐ Sat down (talking, reading, doing schoolwork)

☐ Stood around or walked around

☐ Ran or played a little bit

☐ Ran around and played quite a bit

☐ Ran and played hard most of the time

5. In the last 7 days, on how many days right after school, did you do sports, dance, or play games in which you were very active? (Check one only.)

☐ None

☐ 1 time last week

☐ 2 or 3 times last week

☐ 4 times last week

☐ 5 times last week

6. In the last 7 days, on how many <u>evenings</u> did you do sports, dance, or play games in which you were very active? (Check one only.)

☐ None

☐ 1 time last week

☐ 2 or 3 times last week

☐ 4 or 5 times last week

☐ 6 or 7 times last week

7. <u>On the last weekend</u>, how many times did you do sports, dance, or play games in which you were very active? (Check one only.)

☐ None

☐ 1 time last week

☐ 2 or 3 times last week

☐ 4 or 5 times last week

☐ 6 or more times last week

8. Which one of the following describes you best for the last 7 days? Read all five statements, and encircle the one answer that describes you best.

A. All or most of my free time was spent doing things that involve little physical effort.

B. I sometimes (1 — 2 times last week) did physical things in my free time (e.g. played sports, went running, swimming, bike riding, did aerobics).

C. I often (3 — 4 times last week) did physical things in my free time.

D. I quite often (5 — 6 times last week) did physical things in my free time.

E. I very often (7 or more times last week) did physical things in my free time.

9. Mark how often you did physical activity (like playing sports, games, doing dance, or any other physical activity) for each day last week.

Monday	None ☐	Little bit ☐	Medium ☐	Often ☐	Very often ☐
Tuesday	None ☐	Little bit ☐	Medium ☐	Often ☐	Very often ☐
Wednesday	None ☐	Little bit ☐	Medium ☐	Often ☐	Very often ☐
Thursday	None ☐	Little bit ☐	Medium ☐	Often ☐	Very often ☐
Friday	None ☐	Little bit ☐	Medium ☐	Often ☐	Very often ☐
Saturday	None ☐	Little bit ☐	Medium ☐	Often ☐	Very often ☐
Sunday	None ☐	Little bit ☐	Medium ☐	Often ☐	Very often ☐

10. Were you sick last week, or did anything prevent you from doing your normal physical activities? (Check one.)

☐ NO

☐ YES

If YES, what prevented you? ________________________________

End of the questionnaire
Thank you for completing!

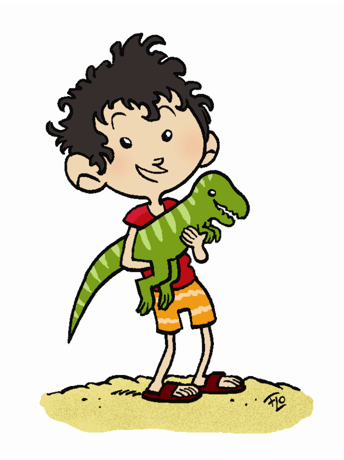

Supplement: Additional file 1 — PA Questionnaire. Physical Activity Questionnaire. Physical Activity Questionnaire as used in the study, based on the Physical Activity Questionnaire for Older Children (PAQ-C) and Children’s Leisure Activities Study Survey (CLASS), cross-culturally adapted to represent common Dutch physical activities. [file 1471-2431-13-102-S1.pdf]
